# The Present Fate of the British Nurse?

**Published:** 1919-06-07

**Authors:** 


### LEST WE FORGET!

### The Present Fate of the British Nurse?

The Red Cross, as the world knows, is the symbol of help for the wounded in war, and one of the essential features of Red Cross achievement—nay, the prime essential—is nursing. This is a truism scarcely necessary to chronicle, but I take the liberty of chronicling it lest we forget. The war is virtually at an end, and the Red Cross is in process of demobilisation. In the midst of rejoicing at the advent of peace the disappearance of the Red Cross worker is an outstanding cause for regret. In the horror and tragedy and sorrow there was present throughout the angel of healing. Frightfulness was strangely intermingled with heroism, devotion, and love. Shot and shell; pain, bloodshed, and death; white crosses to mark the place where heroes sleep. This on the one hand, and on the other armies of British women mobilised of their own free will to watch and wait and bring comfort and balm to the fallen.

It is a familiar tale, but there are chapters of it that may be forgotten in the ecstacy of peace. May I be pardoned for dropping the inquiry—what of the nurse? The public memory is short. For a while there was something heard of the nation's tribute to nurses. Is it being relegated to silence? Is the war nurse already a back number? I confess that so far as I am concerned I have heard very little of the subject of late, and I have scanned the horizon for many weeks. We live in a film age. Wondrous are the reels that dazzle the gaze of the multitude. Yesterday it was the man who went forth to die. To-day it is the man who goes forth to fly. Then the band plays and we have a new film. But no film, however startling, can obscure the fact that we have our debts to pay, and none can deny that the nation owes a huge debt to the British nurse. This is irrecoverable by process of law: it is a debt of honour, and what I have to point out now in plain terms is that the bill has not yet been presented. It is even doubtful if it has been properly made out.

Mark it well, I am not talking of charity. There is no expression that so jarred throughout the war as "war charities," the crowning scandal being that it is legalised and embodied in Acts of Parliament. Who can blame the innocent contributor of five shillings or five pounds for regarding himself as a donor of charity when the State so describes his gift? A man stands in the trenches in defence of the homeland, and the non-fighting civilian, trembling lest he should fail, pays tribute towards his comfort. This act is described in identical terms with that of giving a penny to a medicant. It would seem as though we had lost not merely a sense of proportion but a sense of common decency. Pardon this outburst. My desire is to bring the actual realities of the situation into view. Wherever the fighting man went, thither also went the nurse, and of the millions of brave men who stood between us and destruction, the great majority at some time or other, for great causes or small, benefited from her ministrations. Every wounded soldier is a living testimony to the intrinsic value of the war nurse to the nation's weal. And what is a war nurse? I doubt if there is a trained nurse in the country who is not. The majority of nurses tended soldiers in home hospitals or abroad; and all of them worked at high pressure resulting from the war. Some of them fell at the post of duty, bombed by enemy airmen and gunners. Some of them sleep in the sea. Others are broken and will toil no more.

Eaten bread, they say, is soon forgotten. Have we forgotten, or are we forgetting? The answer, I am afraid, is not in the negative. The Nation's tribute, so far, is little more than words. There is as yet no nation's tribute in actual fact. A few have contributed handsomely, generously, but the Nation—no. There were ten lepers cleansed, but where are the nine? When the nation's tribute is paid, if it is ever paid, every household in the kingdom will have personal and practical experience and knowledge of the fact.

The outlook of the British nurse to-day is precarious. No national pension awaits her disablement. It is true that she is pictured and praised and haloed, but of material benefit she is bereft. It is a poor guerdon for sacrifice and devotion. In a word it is not British, and the sooner this fact is appreciated the better. The nation's tribute is the nation's debt, and it must be honoured. A round million is a modest minimum estimate, a sum which compares unfavourably in every respect with the tribute paid to Florence Nightingale after the Crimean campaign. Such a sum carefully administered, and possibly augmented by nurses themselves, would go a long way towards freeing them from embarrassment and removing the haunting fear of future poverty.

The promoters of this admirable movement need have no fear that the nation will fail in its duty. They need have no fear of the brawlers who object, well knowing that the inspiring motive of the objectors is jealousy, not justice. The objectors must not be permitted to drive the nurse to the wall just because they did not inaugurate the tribute. In a vigorous and thorough campaign of propaganda the Red Cross workers of the Kingdom will co-operate. So will the fighting-men, mobilised and otherwise, including, especially, the wounded. From the pulpits, in the schools, in the workshops, in the homes of the people, if the gospel is preached the response will be whole-hearted and generous. I have faith in my countrymen. The story of the sacrifice of Nurse Cavell is burned into every British heart, and that sacrifice will bring forth fruit a thousandfold.

**But the claim must be made now. To-morrow will be too late.**

IERNE.

